# The Expression of *ARMCX1* in Gastric Cancer Contributes to Prognosis and Influences Chemotherapy

**DOI:** 10.1155/2023/2623317

**Published:** 2023-01-23

**Authors:** Changheng Lv, Di Yang, Donghu Zhang, Jiajia Shen, Zechen Wang, Siyuan He, Lingzhang Meng, Jian Song, Jingjie Zhao

**Affiliations:** ^1^Center for Systemic Inflammation Research (CSIR), School of Preclinical Medicine, Youjiang Medical University for Nationalities, Baise, Guangxi Province, China; ^2^Department of Pediatric Surgery, The Affiliated Hospital of Youjiang Medical University for Nationalities, Baise, Guangxi Province, China; ^3^Institute of Cardiovascular Sciences, Guangxi Academy of Medical Sciences, Nanning, Guangxi Province, China; ^4^Life Science and Clinical Research Center, The Affiliated Hospital of Youjiang Medical University for Nationalities, Baise, Guangxi Province, China

## Abstract

The altered expression of *ARMCX1* in patients with gastric cancer has been reported frequently, yet its correlation to prognosis and chemotherapy needs to be unveiled. In combination of the gene expression data retrieved from TCGA database and bioinformatic analysis, this study discovered 590 differentially expressed genes in the cancerous biopsies isolated from gastric patients, compared with controls. Among which, *ARMCX1* exhibited great potential to serve as a prognostic biomarker for gastric patients; furthermore, patients with low expression of *ARMCX1* could be more sensitive to these 9 chemotherapeutic agents: A-770041, AMG-706, ATRA, BEZ235, bortezomib, CGP60474, dasatinib, HG-64-1, and pazopanib, rather than the other chemotherapeutic agents. This study helps the improvement of evaluating the prognosis of gastric cancer patients, and would help optimize chemotherapeutic strategies in consideration of the expression of *ARMCX1*.

## 1. Introduction

Gastric cancer (GC), or stomach cancer, was listed among the top common cancers throughout the whole world [1–3] and ranks sixth in cancer incidence but second in mortality [4]. The World Health Organization estimated over one million new patients and 769,000 death reports from GC only in 2020, with the highest incidence rates in Asian countries such as Japan, Mongolia, and China [5]. The various treatment options for GC include surgery, radiotherapy, chemotherapy, gene therapy, immunotherapy, and multidisciplinary therapeutic strategy, though help improve the therapeutic outcomes as reported [6], GC remains a major cause of cancer mortality worldwide, with above 700,000 deaths annually [7]. Thus, it is vital to determine the molecular mechanisms that lead to GC, to improve the therapeutic effect.

The gene “ARM Protein Lost In Epithelial Cancers On Chromosome X 1 (*ARMCX1*)”, also represented as *ALEX1*, locating at chromosome region Xq21.33-q22.2, contributes to tumorigenesis [8]. *ARMCX1* has a role in regulation of mitochondrial transport especially in the process of neuronal repair [9]. Recent studies have shown that *ARMCX1* is downregulated in epithelial cancer [10]. Its protein could significantly inhibit the occurrence of GC via affecting the PAR-1/Rho GTPase pathway [11]. Thus, researchers have found *ARMCX1* to be a biomarker to predict the prognosis of GC, and this protein could serve to increase the therapeutic effects.

Therefore, this study is aimed at comprehensively delineating the expression profile of *ARMCX1* in GC patients, to clarify its associations with clinical data and patient prognosis, as well as the potential mechanisms by which *ARMCX1* affects GC progression. Overall, this study identified the relationship between *ARMCX1* and its role in regulating the microenvironment in GC, and evaluated its role in affecting chemotherapy.

## 2. Materials and Methods

### 2.1. GC Data Collection

The expression of *ARMCX1* in various cancer patients was analyzed with TIMER, an internet-based tool for comprehensive analysis of immune phenotypes [12, 13]. The GDC data portal was used to download the FPKM gene expression matrix of GC patients in the TCGA database. The TCGA GC cohort dataset consisted of 435 GC patients and 35 normal tissues. All clinical data and demographic information were checked and filtered from the TCGA portal using the UCSC Cruz Xena functional genomics explorer.

### 2.2. Differences in *ARMCX1* mRNA and Protein Expression between GC and Normal Biopsies

*ARMCX1* mRNA expression in GC was analyzed with limma package in the R Software (version 4.1.2) [14], and with R packages, ggplot2 and pheatmap. The HPA database was employed to validate *ARMCX1* protein expression levels in GC through immunohistochemistry [15, 16].

### 2.3. Flow Cytometry Analysis

Gastric cancerous tissues were isolated from patients by surgery, the paracarcinoma tissues were used as controls in this study. The freshly isolated tissues were digested with Collagenase IV (Gibco, #17104) according to manufacturer's protocols. After resuspension with PBS containing 0.5% BSA, the cells were blocked for unspecific bindings, then the cells were incubated with FITC *ARMCX1* (Biorbyt, #orb102105) on ice for 15 minutes. After washing twice, the cells were recorded on a ThermalFisher Attune Nxt machine. The debris were removed by counterstaining with propidium iodide.

### 2.4. Correlation between *ARMCX1* Expression and Clinical Information

Based on the mean values, patients with GC were grouped into low group with low expression of ARMCX1, and another group with high expression. The clinical data including pathological patterns, clinical stage, and information from follow-up study of GC patients, were assessed, and the association between *ARMCX1* and the clinical pathological data was analyzed.

### 2.5. Survival Analysis of *ARMCX1* in GC

Overall survival (OS), together with progression-free survival (PFS) were calculated with the Kaplan–Meier (KM) method. Univariate and multivariate methods were used to perform prognosis analysis.

### 2.6. Identification of DEGs, GSEA Analysis

Patients/clinical data were stratified into two groups based on the *ARMCX1* expression. DEGs were calculated between the high and low *ARMCX1* expression level. A *p* value lower than 0.05 and ∣logFC∣ value higher or equal to 1 were taken as significant statistically. In addition, the R packages “clusterProfiler,” “http://org.Hs.eg.db,” “enrichplot,” and “ggplot2” were used to conduct GO and KEGG enrichment analyses on the DEGs. GSEA was employed to evaluate the pathways and molecular mechanisms involved in the development of GC [17].

### 2.7. Analysis of the Correlation between *ARMCX1* Expression and the Immune Microenvironment of GC

We used the ESTIMATE algorithm to acquire immune score and stromal score in tumor microenvironment of GC patients, revealing the correlation with *ARMCX1* expression. In addition, we utilized the CIBERSORT method to investigate immune cell infiltration features in GC. The tumor mutation burden (TMB) within a particular region was used for correlation test by Spearman's rank analysis.

### 2.8. Sensitivity Analysis of Chemotherapeutic Agents

The R package pRRophetic was used to explore the sensitivity of chemotherapeutic agents. In the present study, the IC50 values of the most common chemotherapy drugs were calculated using the R packages pRRophetic and ggplot2 to assess clinical responses to treatment.

### 2.9. Statistical Analysis

The *x*^2^ test was used to compare the clinical parameters between the groups glassified by the high and low expression of *ARMCX1*. Survival analysis was performed using the KM method. For determining the independent prognostic factors, we performed both univariate and multivariate analyses. The Spearman method was applied for correlation analysis. For all analyses, *p* value lower than 0.05 was taken as significant. In all the figures shown in this study, the asterisks indicate statistically significant differences (^∗^ refers *p* < 0.05; ^∗∗^ refers *p* < 0.01, and ^∗∗∗^ refers *p* < 0.001).

## 3. Results

**Figure 1:**
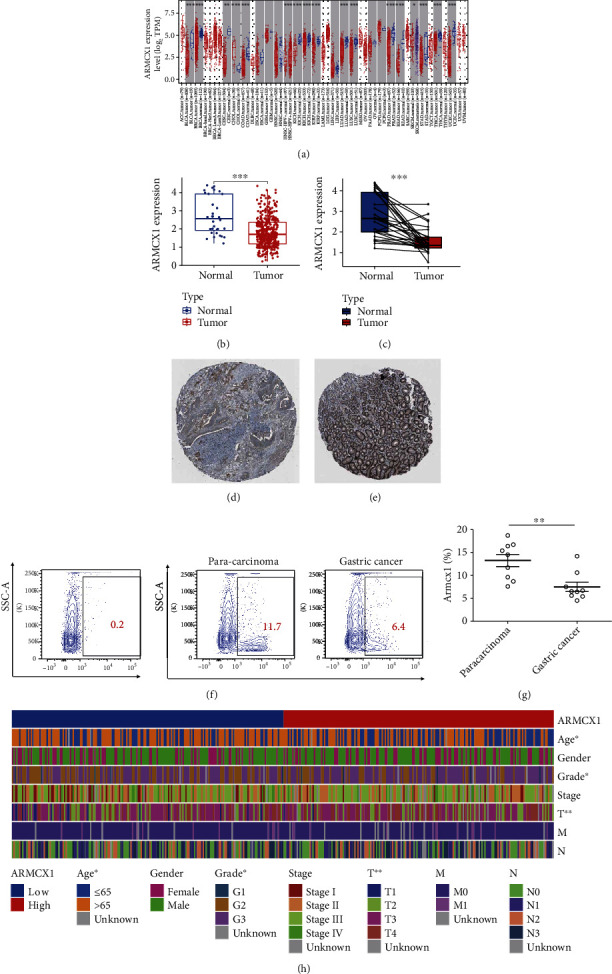
GC biopsies exhibited lower expression of *ARMCX1*. (a) *ARMCX1* expression in various cancerous tissues. (b, c) *ARMCX1* expression in The Cancer Genome Atlas database compared with that in normal tissue. (d, e) Immunohistochemistry staining showed *ARMCX1* protein in GC tissue. (f) Contour plots showed the expression of *ARMCX1* in gastric cancer biopsies compared with paracarcinoma controls. (g) Statistical analysis of the expression level of *ARMCX1* in gastric cancer biopsies compared with paracarcinoma controls. ^∗∗^*p* < 0.01. Each dot represents one readout. Nonparametric test. (h) Correlation between *ARMCX1* and clinicopathological data of patients with GC.

The analysis with TIMER showed that *ARMCX1* was drastically lower in various cancerous tissues, such as malignant bladder, breast, uterine neck, colon, kidney, lung, prostates, stomach, and thyroid; while *ARMCX1* expression was higher in cholangiocarcinoma (Figure 1(a)). Besides, we noticed from the TCGA cohort dataset that the gene *ARMCX1* was drastically lower in GC than in normal tissues (Figures 1(b) and 1(c)). Furthermore, the HPA portal showed that *ARMCX1* protein was lower in GC than in controls, especially when compared to that in adipose tissue (Figures 1(d) and 1(e)). In consistence, flow cytometry analysis of gastric cancerous biopsies showed *ARMCX1* is significantly lower than in paracarcinoma controls (Figures 1(f) and 1(g)).

Correlation test between two groups (one group contained GC patients with high expression of *ARMCX1,* another contained low expression) exhibited that *ARMCX1* expression was correlated with age, Grade, and T; however no significant correlation with sex, stage, M, and N (Figure 1(h)).

**Figure 2:**
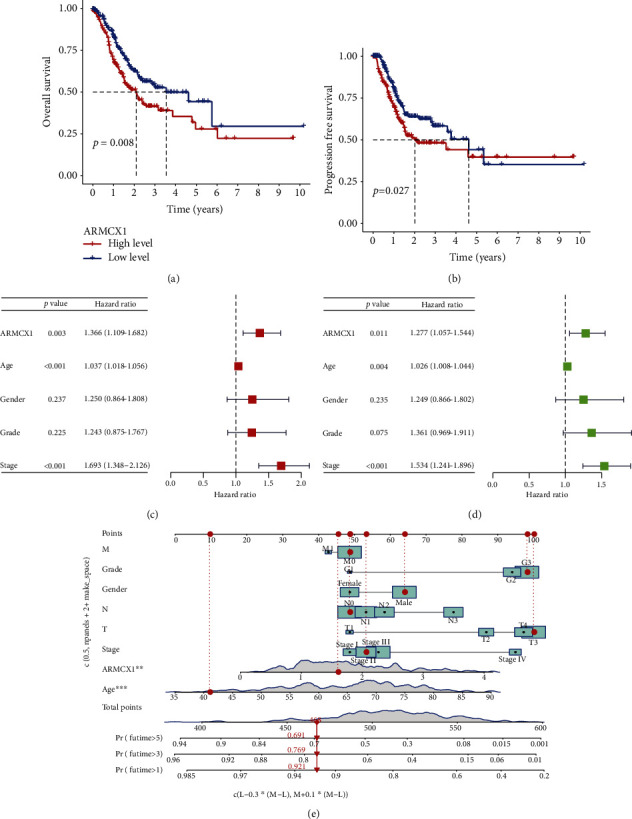
*ARMCX1* expression could potentially serve as a prognostic marker. (a) Correlation between *ARMCX1* expression and overall survival (OS). (b) Correlation between *ARMCX1* expression and PFS. (c) Multivariate analysis of overall survival. (d) Univariate analysis of overall survival. (e) The total nomogram point of each patient can be used to predict the probability of survival at 1, 3, and 5 years.

KM analysis showed individuals with high *ARMCX1* expression could have a worse OS (*p* = 0.008), while patients with low *ARMCX1* expression had nice FDS value (*p* = 0.027) (Figures 2(a) and 2(b)). Univariate analysis indicated that *ARMCX1* expression (*p* = 0.003), age (*p* < 0.001), and stage (*p* < 0.001) were prognostic factors (Figure 2(c)). Furthermore, *ARMCX1* showed a great potential to predict the survival of GC patients, based on multivariate analysis (*p* < 0.011) (Figure 2(d)). Such an outcome indicates *ARMCX1* could serve as an independent biomarker for prognosis.

Based on which, we built a nomogram that could be used for the prediction of 1-year, or 3-year, and or 5-year OS for GC patients (Figure 2(e)).

**Figure 3:**
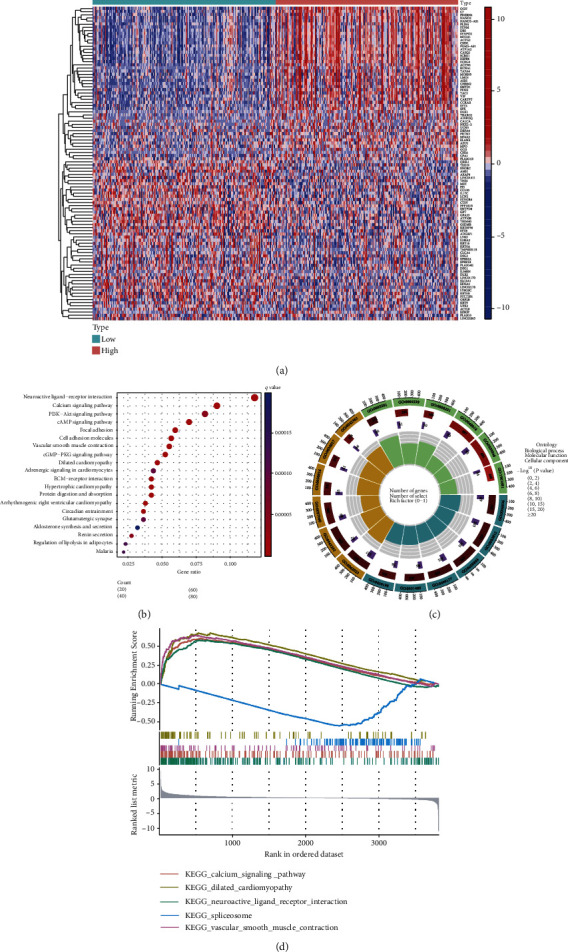
Pathway analysis between high and low expression of *ARMCX1* in GC patients. (a) Heat maps of the DEGs between the high and low *ARMCX1* expression groups. (b) Bubble diagram of Kyoto Encyclopedia of Genes and Genomes mechanism analysis. (c) GO analysis of the DEGs between the high and low *ARMCX1* expression groups. (d) Significant pathways identified by GSEA analysis.

Depending on the median expression of *ARMCX1*, patients were classified as having either high or low *ARMCX1* levels. 590 DEGs were identified between these two groups (Figure 3(a)). Additionally, we performed a KEGG pathway analysis, and the results are shown as a bubble chart (Figure 3(b)). A circular chart depicts GO analysis of DEGs (Figure 3(c)). GSEA revealed that 22 KEGG pathway-related gene sets were enriched, and a sample GSEA enrichment plot is shown (Figure 3(d)).

**Figure 4:**
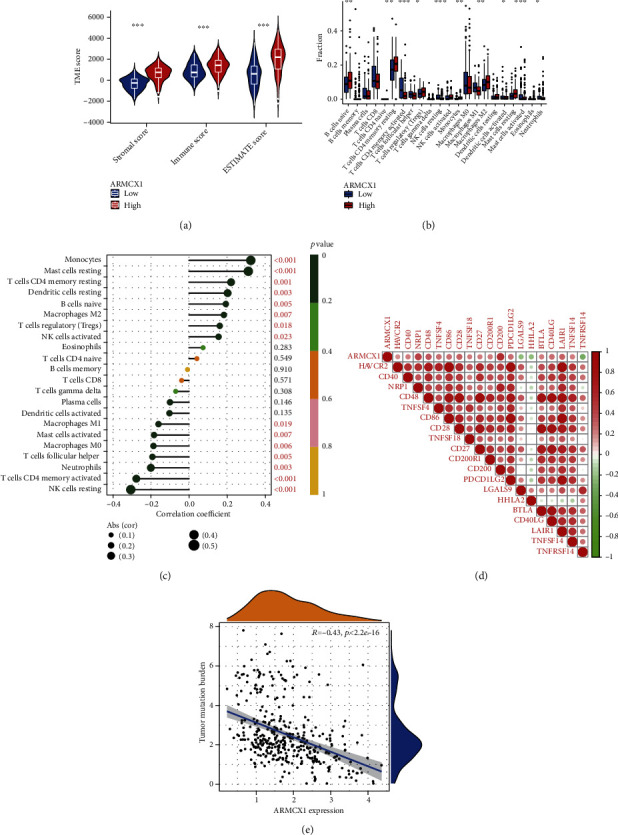
Comparison of tumor microenvironments between GC patients with high and low *ARMCX1* expression. (a) Difference in tumor microenvironment score between the high and low *ARMCX1* groups. (b) Immune infiltration by 22 immune cell types in patients with GC exhibiting high and low *ARMCX1* expression. (c) Correlation between *ARMCX1* gene expression and immune cell infiltration. (d) Correlation of *ARMCX1* expression with immune checkpoint gene expression. Red and green represent positive and negative correlations, respectively. (e) Correlations between *ARMCX1* gene and tumor mutation burden (*p* < 2.2e − 16, *R* = −0.43).

Furthermore, we compared the stromal, immune, and ESTIMATE scores between low-/high-*ARMCX1* expression groups. It showed that high *ARMCX1* expression showed significantly higher immune, stromal, and ESTIMATE scores (Figure 4(a)). Naïve B cells, resting CD4 memory T cells, monocytes, M2 macrophages, and resting mast cells exhibited higher expression, while activated CD4 memory T cells, follicular helper T cells, resting NK cells, M1 macrophages, and neutrophils were expressed at lower levels in the high *ARMCX1* group than in the low *ARMCX1* expression group (Figure 4(b)). Correlation analysis revealed that *ARMCX1* expression was significantly positively associated with monocytes, resting mast cells, resting CD4 memory T cells, resting dendritic cells, naïve B cells, M2 macrophages, regulatory T cells (Tregs), and activated NK cells. Additionally, M1 macrophages, activated mast cells, M0 macrophages, follicular helper T cells, neutrophils, activated CD4 memory T cells, and resting NK cells were negatively correlated with *ARMCX1* expression (Figure 4(c)). Next, we analyzed the correlation between *ARMCX1* and multiple immune checkpoints, which is illustrated in Figure 4(d). Finally, we calculated the TMB for each GC tumor sample. *ARMCX1* expression was negatively correlated with TMB in GC patients (*p* < 2.2e − 16, *R* = −0.43) (Figure 4(e)).

**Figure 5:**
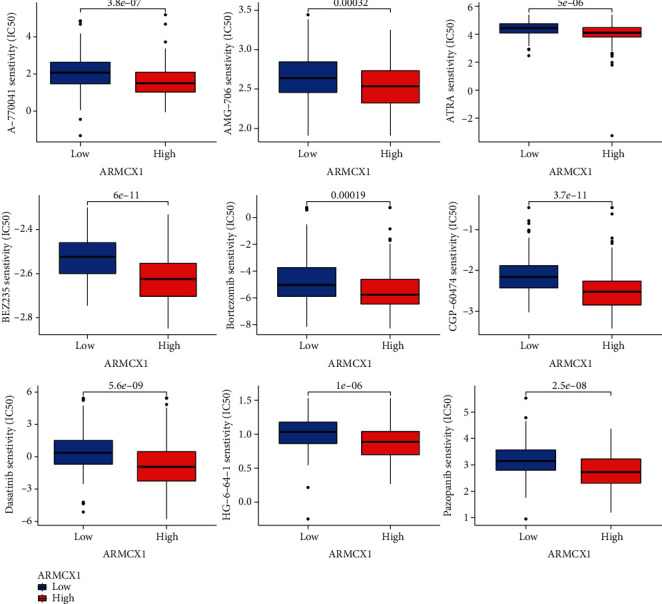
Influence of *ARMCX1* on chemotherapy against GC. *ARMCX1* influenced chemotherapy induced by different drugs in GC patients.

To better understand the differences in drug sensitivity between high and low-*ARMCX1* groups, we performed a GDSC drug sensitivity analysis using 61 different chemotherapeutic agents and found differences in drug sensitivity between these two groups. The top nine results showed that A-770041 (*p* = 3.8e − 07), AMG-706 (*p* = 0.00032), ATRA (*p* = 5e − 06), BEZ235 (*p* = 6e − 11), bortezomib (*p* = 0.00019), CGP-60474 (*p* = 3.7e − 11), dasatinib (*p* = 5.6e − 09), HG-64-1 (*p* = 1e − 06), and pazopanib (*p* = 2.5e − 08) exhibited better sensitivity in the low *ARMCX1* expression group (Figure 5).

## 4. Discussion

Using a pancancer analysis of TIMER databases, we examined the relationship between *ARMCX1* and multiple cancer tumorigenesis models. We also determined the clinical significance of *ARMCX1* in GC progression using the TCGA database. Both results showed that *ARMCX1* levels were lower in GC than in normal tissue. Using the HPA database, Armcx1 protein was also lower in GC tissue compared to that in normal tissue. Clinical data showed that low *ARMCX1* was correlated with OS and PFS and serves as a “solo” prognostic factor. *ARMCX1* has been reported in various human tissues, including lung cancer, prostate cancer, colon cancer, pancreatic cancer, and ovarian cancer [18].

The function of *ARMCX1* is relatively unknown; however, a recent study reported that *ARMCX1* has been linked to the RNA damage response and RNA modification [19]. Similar to our results, Wang et al. revealed that the expression rate of *ARMCX1* was significantly reduced in GC samples compared to that in normal samples [20]. Some studies, however, have yielded conflicting results. Ecker et al. found that in cervical squamous cell carcinoma, the expression of *ARMCX1* protein was significantly increased compared with that in noncancerous tissues [21].

It has been observed that different outcomes from patients receiving chemotherapy, even those patients with the same cancer, for example, GC, but less is known about the molecular mechanism. This paper discovered *ARMCX1* could not only serve as a prognostic marker, but also potentially contribute to chemotherapy by affecting various signals, including regulation of mitochondrial dynamics. To clarify the mechanism by which *ARMCX1* contributes to GC progression, GO annotation, KEGG signaling pathway, and GSEA analyses were performed for the high and low *ARMCX1* expression groups. Here, we found that some classical signaling pathways may play important roles in GC, including the PI3K − Akt, cAMP, calcium, and cGMP−PKG signaling. The PI3K − AKT pathway, an important effector downstream of growth factor receptors, is dysregulated in cancer types [22–25]. It has been proven that PI3K/AKT/mTOR signaling regulates apoptosis and autophagy and constitute a molecular target for cancer therapy [26]. The PI3K/AKT/mTOR signaling pathway is responsible for inhibiting autophagy in human cervical cancer cells by miR-338-3p [27]. Further, RA-induced apoptosis in breast cancer cells is mediated by the PI3K/AKT/mTOR pathway [28]. All of which participated in regulation of mitochondrial stability and dynamics [29–32]. Besides, GC has been associated with abnormal activating RAS initiated signaling [33, 34]. According to previous studies, ASPN also accelerates cell proliferation via PSMD2 and ELK, p38/MAPK, and PI3K/AKT signaling in GC cells [35–37]. But, whether *ARMCX1* could affect these signals needs to be further studied.

In this study, we discovered the expression level of *ARMCX1* in gastric cancer influences the outcome of chemotherapy (a clinical treatment initiated to destroy cancer cells), probably due to *ARMCX1'*s regulation on mitochondrial dynamics [38, 39], an important downsteaming procedure for the induction of cancer cell apoptosis [40, 41].

Moreover, it has been showed that cAMP signaling pathway is vital for the development of bladder cancer metastasis via affecting microtubule [42]. Long noncoding RNA KCNQ1OT1 upregulates the methotrexate resistance of colorectal cancer cells by inhibiting miR-760/PPP1R1B [43]. Elevated epinephrine levels in prostate cancer can inhibit apoptosis and drive tumor growth through the cAMP in murine [44]. The calcium signaling pathway plays an important role in breast cancer development [45]; moreover, calcium signaling is relevant to the proliferation, migration, invasion, and drug resistance of cancer cells [46–48].

The cGMP-PKG signaling pathway has also been linked to colon cancer and proposed as a therapeutic strategy for colon cancer [49]. Cell proliferation and apoptosis are critically affected by the cGMP-PKG pathway, which prevents colon cancer progression [32, 50, 51]. cGMP-PKG plays a central role in fibrotic processes in the liver, and defects in this pathway cause impaired NO-dependent responses in hepatic stellate cells on activation [52]. Burn-induced cardiac mitochondrial dysfunction is modulated via the cGMP-PKG pathway [53]. Overall, pathway network analysis showed that these signaling pathways may play key roles in GC progression.

Over the past decade, the TME has been intensely investigated, especially the immune microenvironment. We calculated TME scores based on the ESTIMATE algorithm to detect the correlations between immune/stromal/ESTIMATE scores and *ARMCX1* expression. We found that the high *ARMCX1* expression group displayed higher stromal scores, immune scores, and estimate scores than the low *ARMCX1* expression group. Similar to the present study, bioinformatics analysis demonstrated that TOX expression is negatively correlated with TumorPurity and positively correlated with ImmuneScore and StromalScore in colorectal cancer [50]. Tumor-infiltrating immune cells are a part of the complex TME, which plays an essential role in tumor progression. Solid cancers are prone to infiltration by immune cells that contribute to tumor progression [51–53].

In this current study, five tumor-infiltrating immune cell types were more prevalent in the high *ARMCX1* expression group, while five other tumor-infiltrating immune cells (Naïve B cells, resting CD4 memory T cells, monocytes, M2 macrophages, and resting mast cells), which were prevalent in the low *ARMCX1* expression group. Among those tumor infiltrating immune cells, memory T cells, monocytes, and M2 macrophages have been extensively studied, however, in this study, the biological/pathological role of the Naïve B cells and resting mast cells in the development of GC, needs to be elucidated.

Correlation analysis revealed that *ARMCX1* expression was significantly positively associated with eight tumor-infiltrating immune cell types, while six other tumor-infiltrating immune cells were negatively correlated with *ARMCX1* expression. Finally, we revealed that *ARMCX1* expression was correlated with immune checkpoints and TMB. Based on the results of this study, the correlations between *ARMCX1* expression and TME score, tumor infiltrating immune cells, immune checkpoints, and TMB may be useful to the development of novel cancer therapies.

Further studies should be performed including the adaptation of *ARMCX1* knockout mouse with GC symptoms to confirm the potential molecular mechanisms proposed by this study, by which, *ARMCX1* affects the prognostic procedure and chemotherapy.

## 5. Conclusion

According to our findings, variations in *ARMCX1* expression levels are associated with GC prognosis. In addition, this study indicated *ARMCX1* was associated obviously with multiple immune signatures. Therefore, the present study provides insights into the protective role of *ARMCX1* in tumor immunology and its potential as a prognostic biomarker for GC.

## Data Availability

The datasets and code generated or analyzed in this study are available from the corresponding author upon reasonable request.
